# *In Silico* Study of Alkaloids as α-Glucosidase Inhibitors: Hope for the Discovery of Effective Lead Compounds

**DOI:** 10.3389/fendo.2016.00153

**Published:** 2016-12-19

**Authors:** Muhammad Zafar, Haroon Khan, Abdur Rauf, Ajmal Khan, Muhammad Arif Lodhi

**Affiliations:** ^1^Department of Pharmacy, Abdul Wali Khan University, Mardan, Pakistan; ^2^Department of Chemistry, University of Swabi, Swabi, Pakistan; ^3^Department of Chemistry, COMSATS Institute of Information Technology, Abbottabad, Pakistan; ^4^Department of Biochemistry, Abdul Wali Khan University, Mardan, Pakistan

**Keywords:** α-glucosidase inhibitors, homology modeling, molecular docking, Molecular Operating Environment, α-glucosidase

## Abstract

α-Glucosidase (extinction coefficient 3.2.1.20) is a primary carbohydrate metabolizing enzyme that acts on the 1–4 associated α-glucose residues. The inhibition of α-glucosidase slows down the process of carbohydrate digestion and avoids postprandial hyperglycemia, which is a major cause of chronic diabetes-associated complication. This study was designed to evaluate the binding capacity of isolated alkaloids with targeted receptor. For this purpose, the three-dimensional tertiary structure of the α-glucosidase was generated by using the Molecular Operating Environment (MOE). The generated model was then validated by using the RAMPAGE and ERRAT server. The molecular docking of 37 alkaloids along with standard acarbose and miglitol reported as a α-glucosidase inhibitor was performed *via* MOE-Dock implemented in MOE software to find the binding modes of these inhibitors. The results showed that compound **17** (oriciacridone F) and **24** (O-methylmahanine) demonstrated marked interaction with active residues and were comparable to standard inhibitors. In short, this study provided computational background to the reported α-glucosidase inhibitors and thus further detail studies could lead to novel effective compounds.

## Introduction

α-Glucosidase is a primary carbohydrate digestive enzyme, which is present in the brush border of the small intestine. It has action on 1,4-α bonds and thus different from β-glucosidase (1–4)^1^. α-Glucosidase catalyzes starch and disaccharides to glucose. Maltase is a similar enzyme that acts upon maltose and is nearly equivalent in function to α-glucosidase. The carbohydrates need metabolism by α-glucosidase before being absorbed into the small intestine. By inhibiting the α-glucosidase, the process of carbohydrate digestion slows down, which helps prevent postprandial hyperglycemia because postprandial hyperglycemia is a major cause of chronic diabetes and associated complications (5). In this regard, several research groups have been working on the desiging of new effective α-glucosidase inhibitors that can be used as therapeutic agents for the suppression of metabolic disorders such as hyperglycemia, obesity, and non-insulin-dependent type II diabetes mellitus (5).

The therapeutic potential of alkaloids has been recognized in the treatment of various human disorders (6–11). The literature review revealed that approximately 37 isolated alkaloids have been tested for α-glucosidase inhibitory activity (12), as shown in Table 1. Gao and colleagues isolated two compounds **1–2** from the leaves of *Adhatoda vasica* Nees. Both these compounds caused marked competitive α-glucosidase inhibition in animal models (13). The phytochemical studies of Campanulaceae *Lobelia* species led to the isolation of 10 compounds **4–12**. These compounds also possessed significant anti-glucosidase effect (14). The potent inhibitors, deoxynojirimycin (DNJ) and 2,5-bis(hydroxymethyl)-3,4-dihydroxypyrrolidine (DMDP) (14–18), were isolated from many plants. The DNJ, along with several other therapeutic effects, provoked outstanding attenuation on glucosidase and thus clinically used as a zero-harm-antidiabetic drug agent. Three more alkaloids named piperumbellactams A–C **13–15** have been isolated from *Piper umbellatum*, produced significant α-glucosidase inhibition (19). Wansi et al. isolated acridones alkaloids **16–18**, from *Oriciopsis glaberrima* Engl, also displayed profound α-glucosidase inhibition, especially compound **17** (oriciacridone F) with IC_50_:34.05 mM (20). *Buthus martensii* Karsch led to the isolation of **19**, a potent non-competitive glucosidase inhibitor (21). Similarly, *Piper sarmentosum* led to the purification of two chaplupyrrolidones alkaloids A **20** and B **21**, which possessed strong anti-glucosidase activity (22). *Murraya koenigii* guide to the isolation of six different alkaloids **22–27** that caused α-glucosidase inhibition. Of the compounds, O-methylmahanine **24** showed marked effect with IC_50_ 29.1 μM (23). Tabussum and co-worker isolated plicatanins A–D **28–31** alkaloids from *Chrozophora plicata* also caused significant α-glucosidase inhibition (24). Six alkaloids **32–36** with potent α-glucosidase inhibitory activity have been isolated from *Morus atropurpurea* (12).

**Table 1:** **The structure of the isolated alkaloids with docking scores**.

S. No.	Structures	Docking score(s)
1	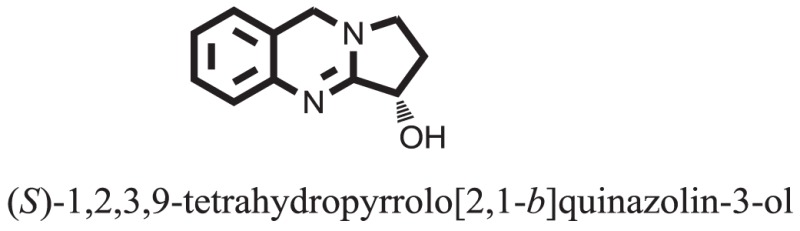	−6.5918
2	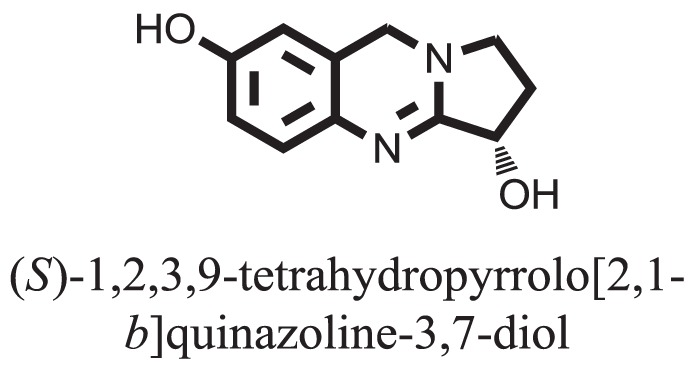	−6.6343
3	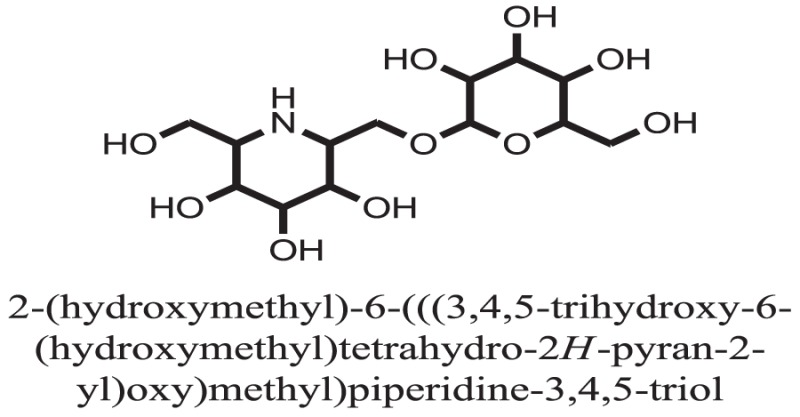	−8.6058
4	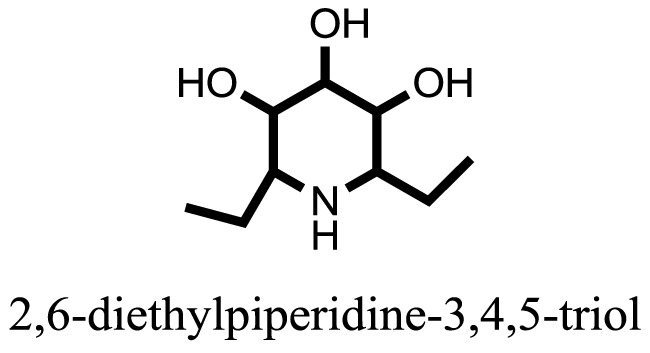	−6.1790
5	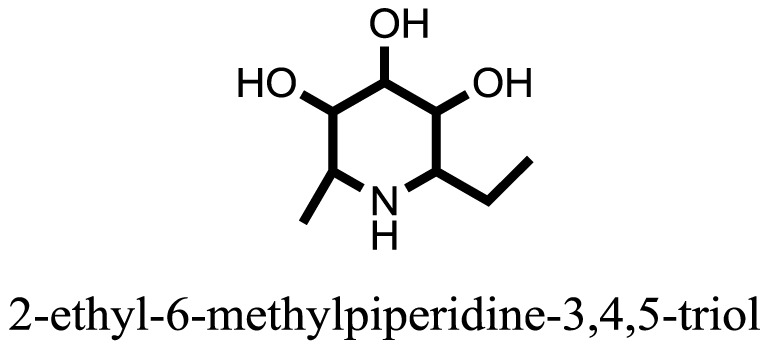	−8.8493
6	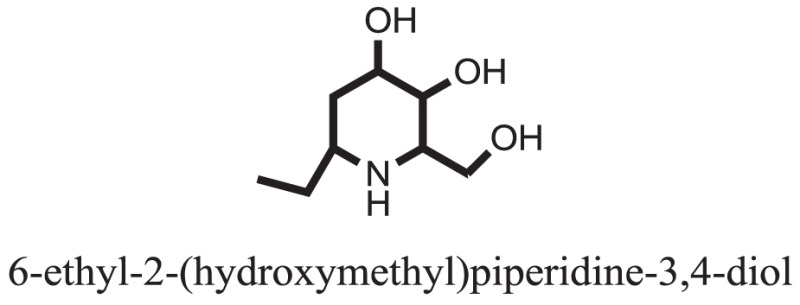	−6.9539
7	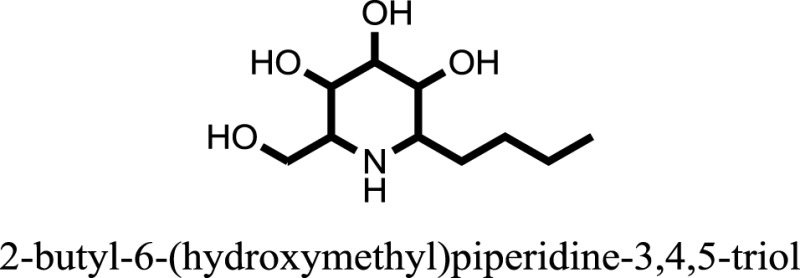	−7.7617
8	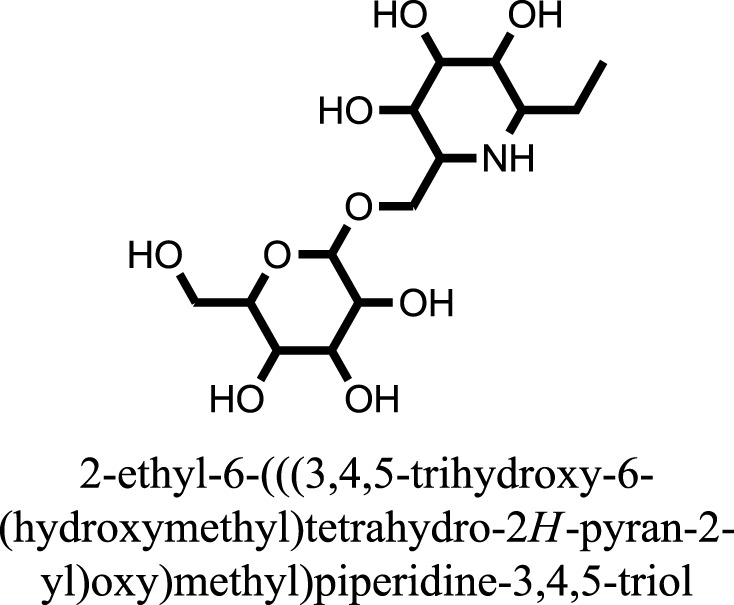	−9.3806
9	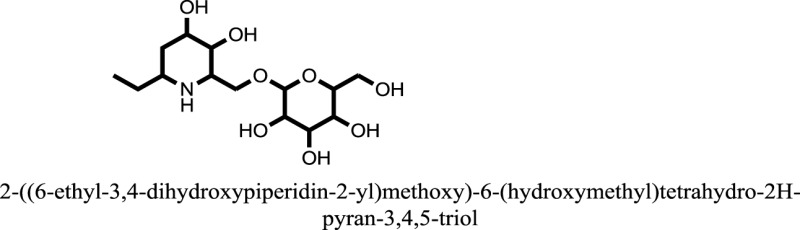	−7.4514
10	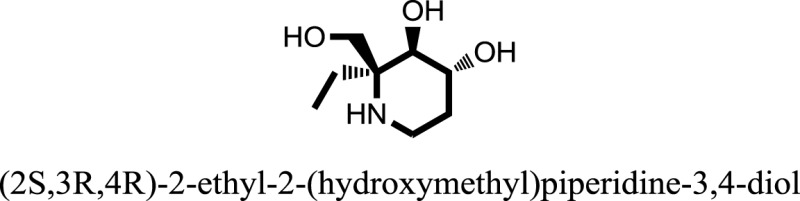	−8.7862
11	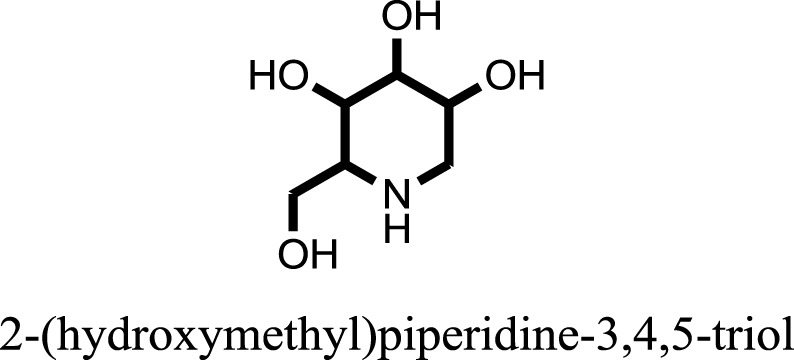	−6.0582
12	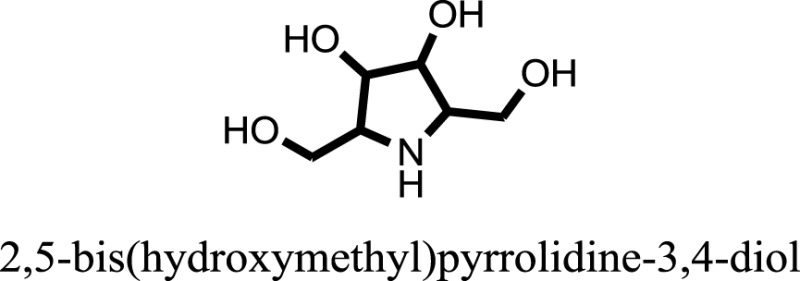	−8.3342
13	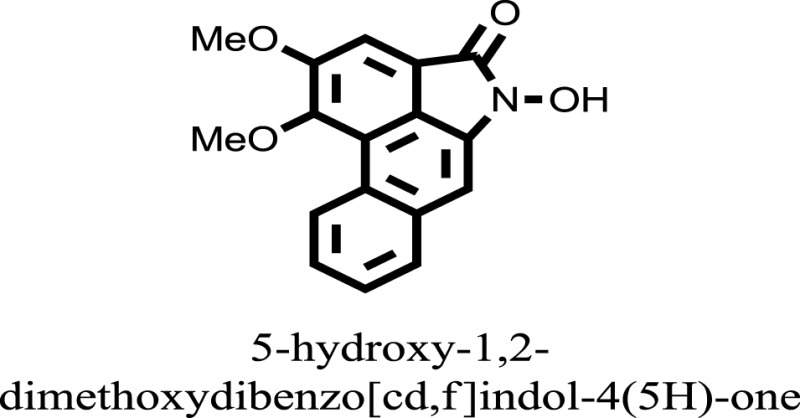	−8.4982
14	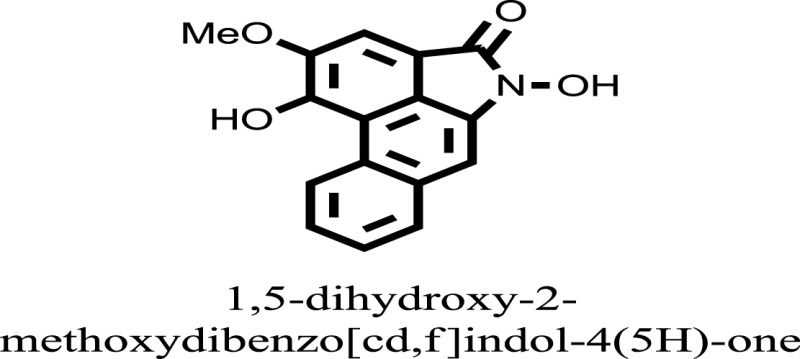	−11.3333
15	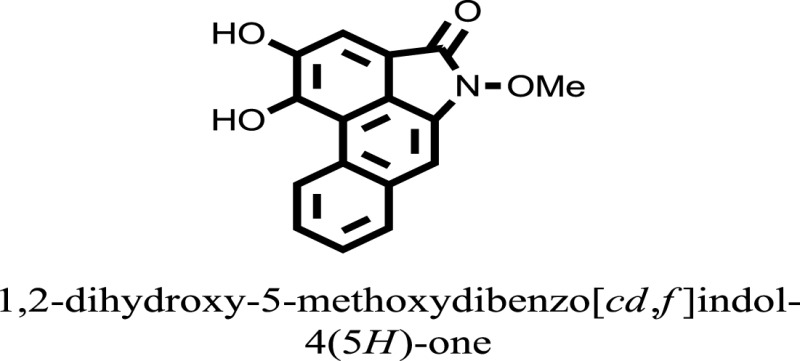	−6.5704
16	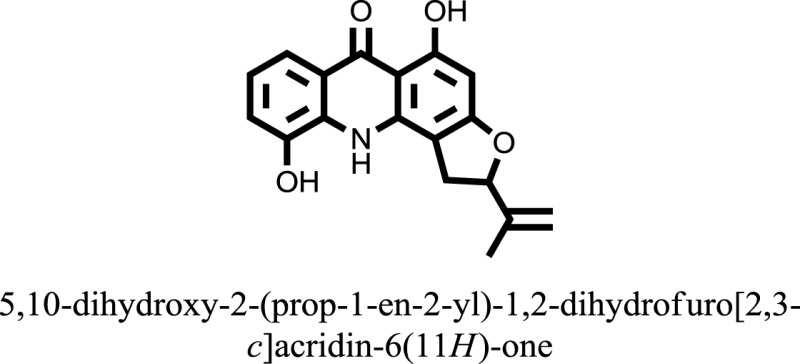	−10.6081
17	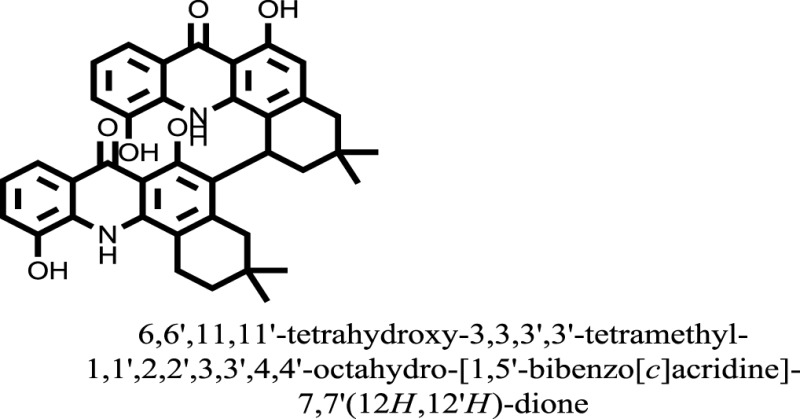	−15.1310
18	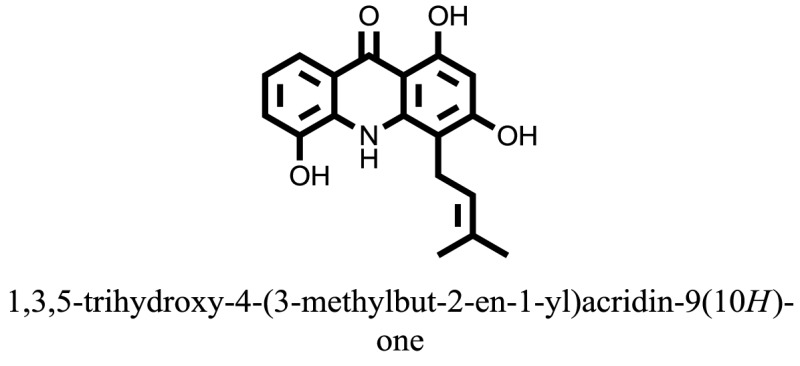	−8.2973
19	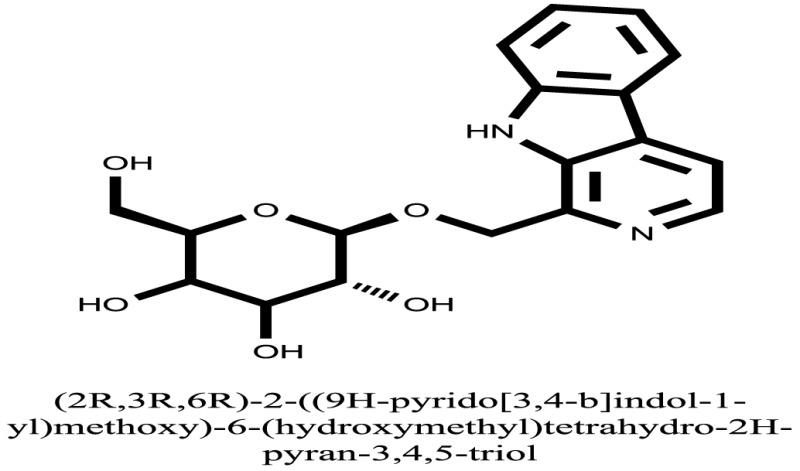	−8.0484
20	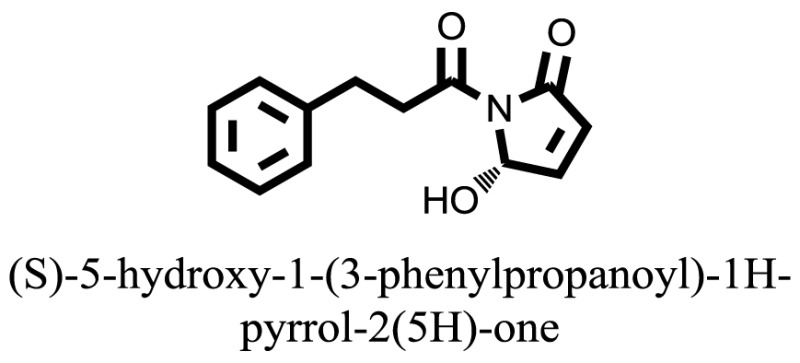	−7.3406
21	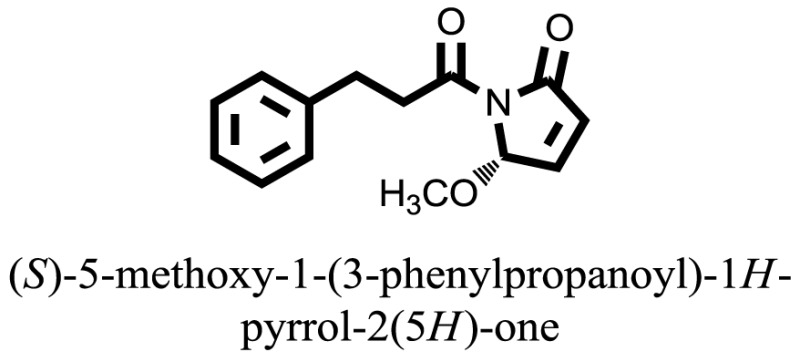	−9.4372
22	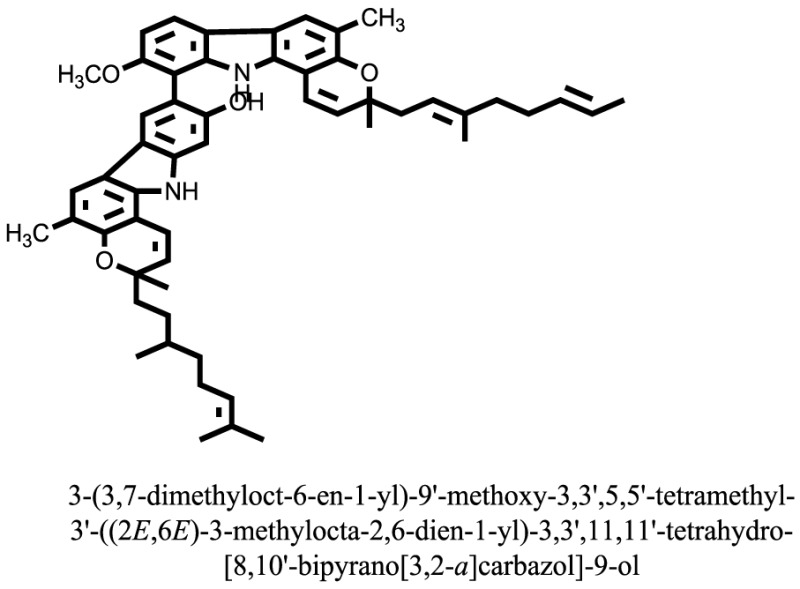	−13.6324
23	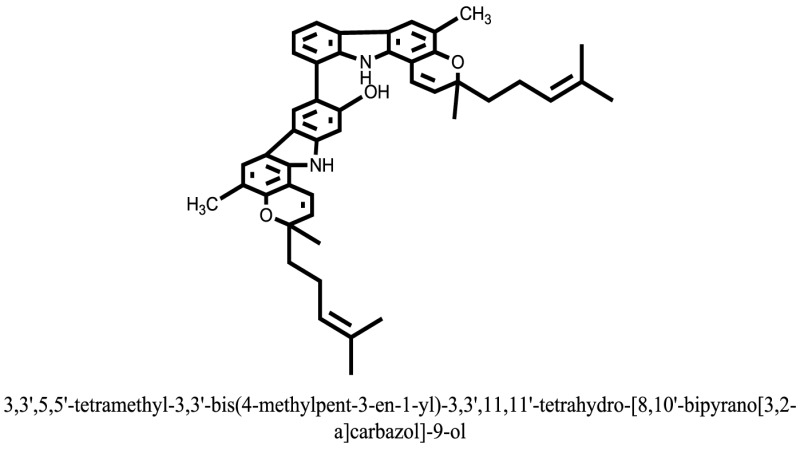	−11.5949
24	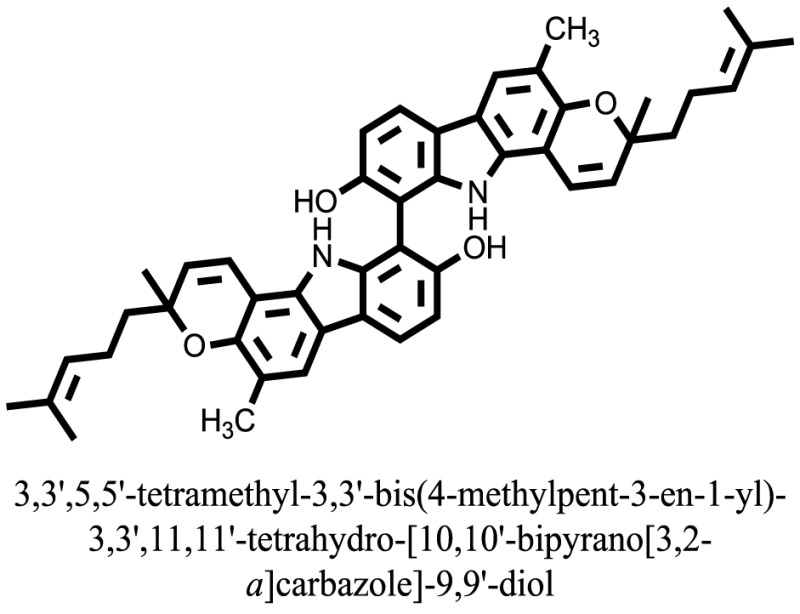	−14.9192
25	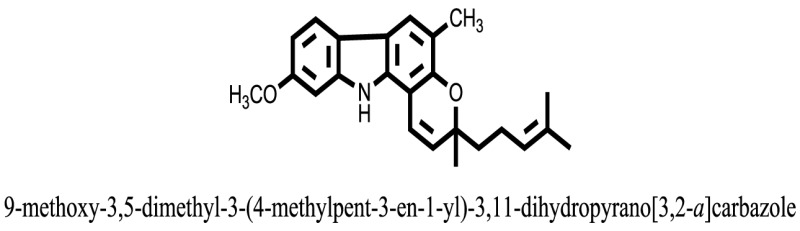	−10.1657
26	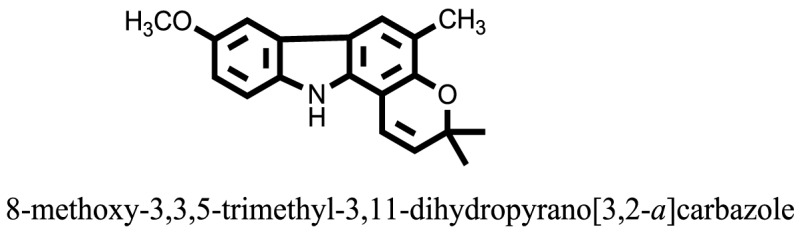	−8.4088
27	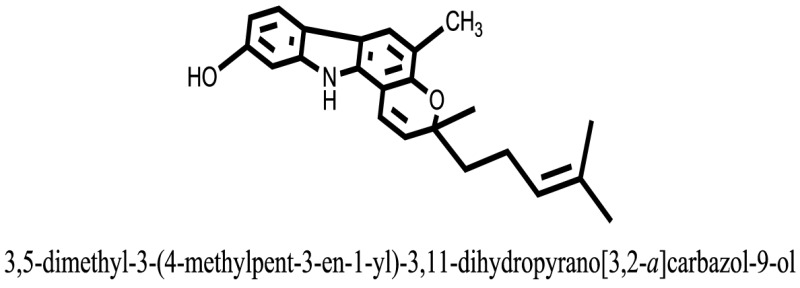	−10.4082
28	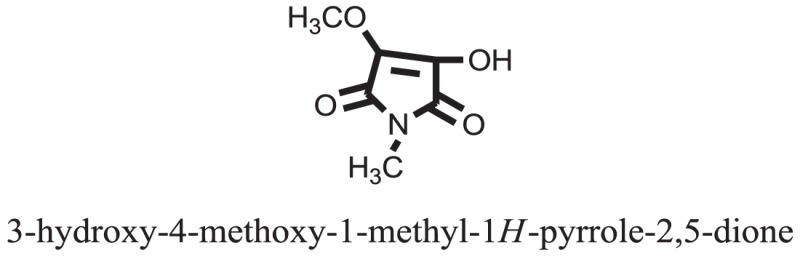	−6.2268
29	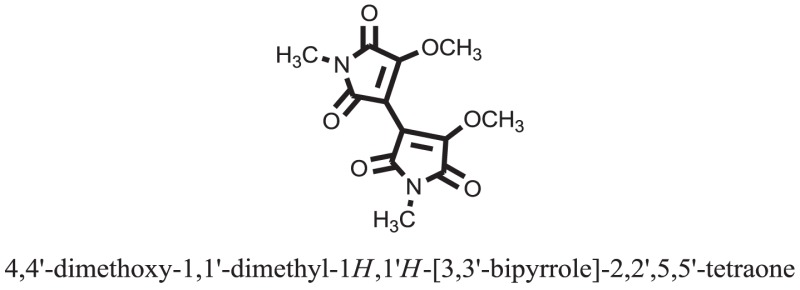	−8.4188
30	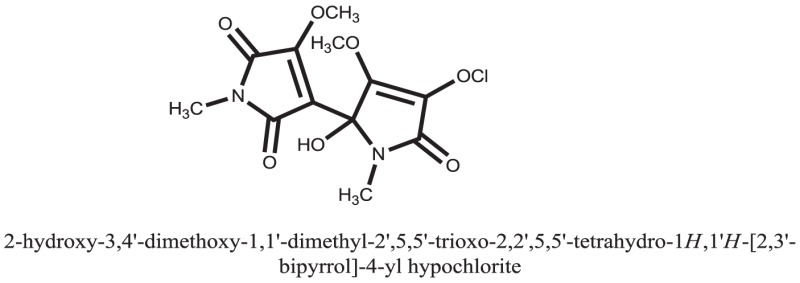	
31	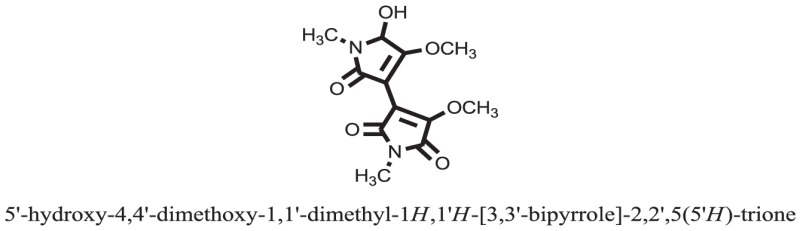	−7.4784
32	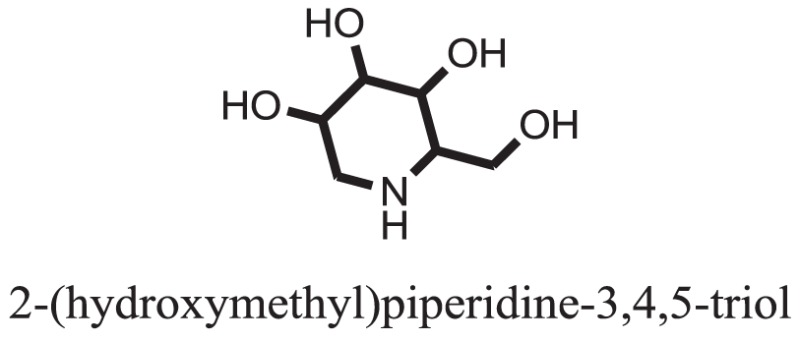	−9.5179
33	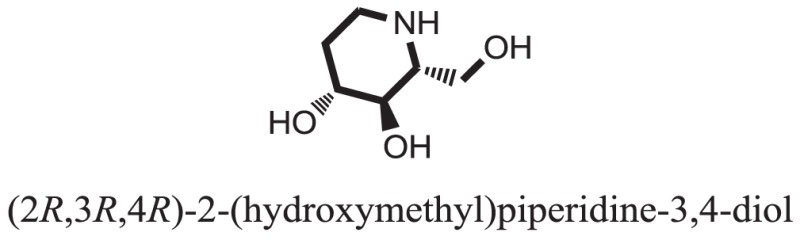	−5.8070
34	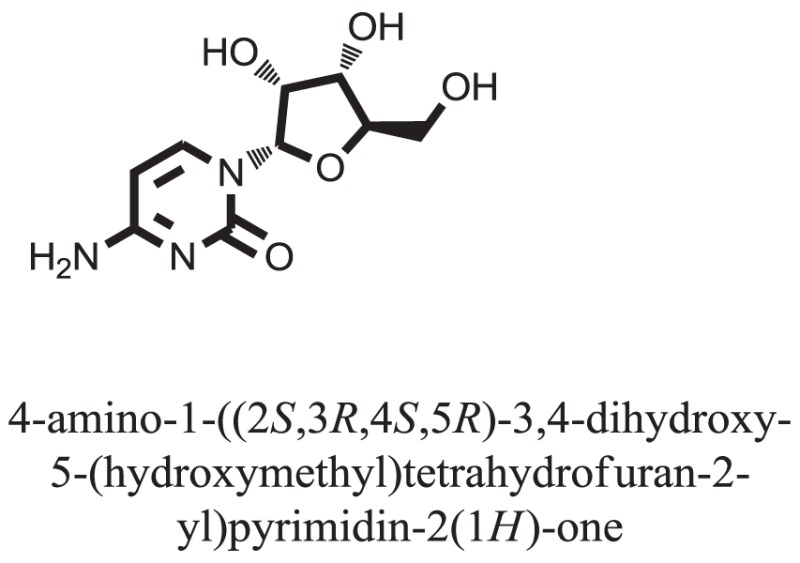	−12.1778
35	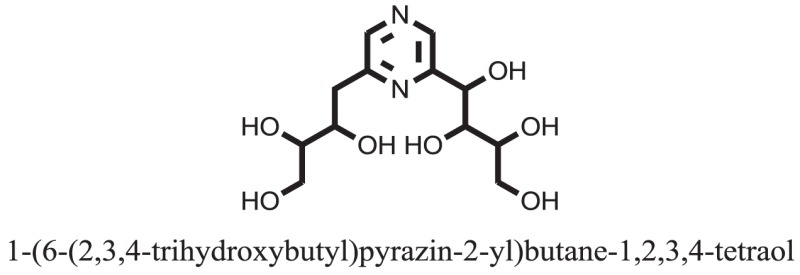	−8.9628
36	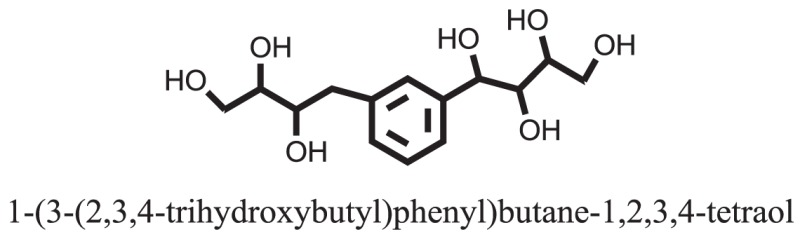	−10.5989
37	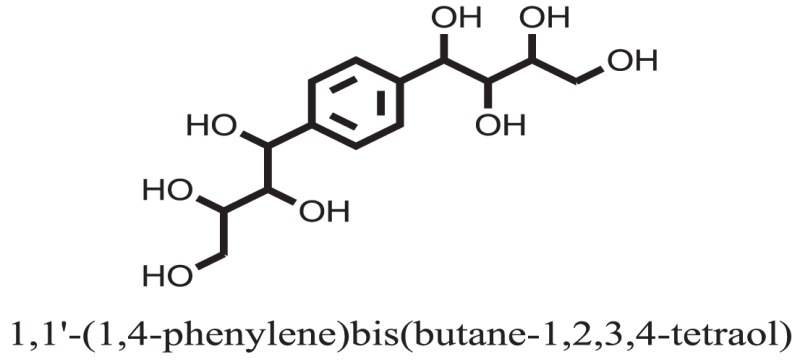	−10.4884
Standard	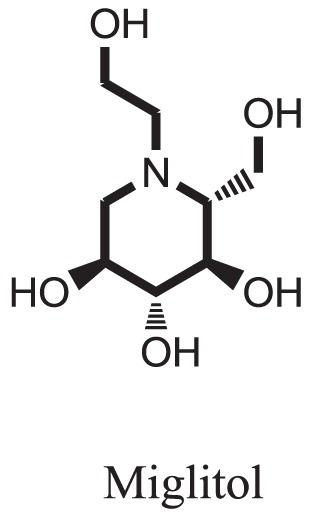	−15.4423
Standard	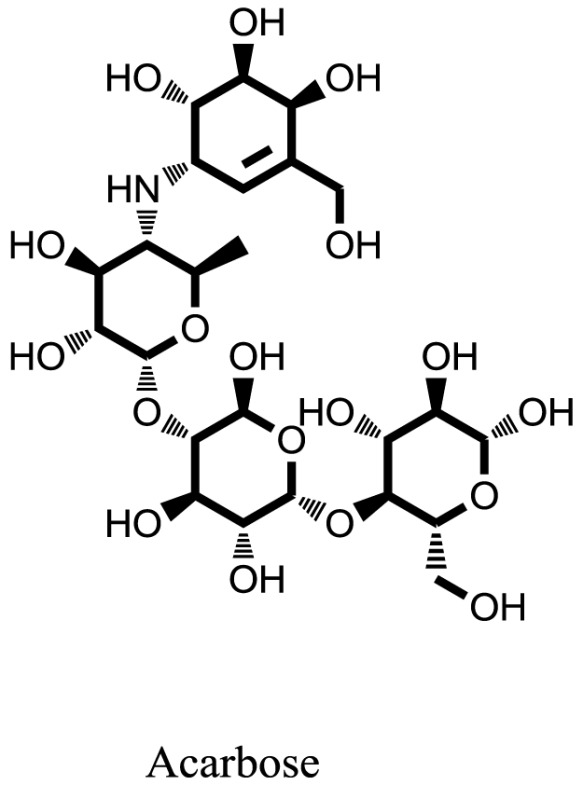	−14.7983

The aim of this research study was to find out the interaction of these reported alkaloids with the target protein and thus findings could be useful for the discovery of new, potent, and active α-glucosidase inhibitors.

## Materials and Methods

In this research studies, we have generated the three-dimensional structure of the glucosidase by using the Molecular Operating Environment (MOE) software, and molecular docking study was performed on the same software. The MOE-Dock was used as docking software implemented in MOE, and ligplot is implemented in MOE for the purpose to visualize the interaction between protein and ligand.

### Retrieval of the Target Sequence

The primary sequence of the α-glucosidase of *Saccharomyces cerevisiae* (Baker’s yeast) was retrieved using Uniprot (Universal Protein Resource)^2^ in FASTAformat, and the target sequence was then kept in the text file for further evaluation. The accession number of α-glucosidase of *S. cerevisiae* is P07265.

### Template Selection

The target sequence of α-glucosidase was downloaded from the Universal Protein Resource (uniprot dataset). Then, Protein-BLAST (25) was done to identify homologs in the PDB (RCSB Protein Databank) (26). Hence, the crystal structure of Isomaltase from the *S. cerevisiae* (Pdb Id: 3A47−A), which has 72% sequence identity to the target protein, was selected as the template for the target protein sequence for the prediction of the tertiary structure of target protein.

### Alignment of the Target-Template Sequence

For the sequence alignment of the target protein, α-glucosidase and template protein (PDB ID: 3A47−A), multiple sequence alignment (MUSCLE) was employed (27) server.^3^ The ClustalW program from the MUSCLE server was used for the alignment of the target-template sequence.

### Homology Modeling

The amino acid sequence of the target protein in FASTA format was copied and paste in the sequence editor of the MOE software. Then, the template protein was loaded in the same MOE software. The chain **1** showed the target protein sequence, and chain **2** showed the template protein sequence. The target and template sequences were aligned before starting the homology modeling and calculated the root mean square deviation (RMSD) with the template. In model refining tool, the intermediate was set to medium, final model to medium, by means of scoring function generalized born/volume integral (GB/VI). Amber 99 with Solvation RField was used as a force field. Various 10 models were formulated, while the final refine model was introduced to MOE main window.

### Validation of Modeled Structure

The overall geometric and stereochemical qualities of the final model were examined using RAMPAGE and ERRAT server (28, 29). ERRAT server was used to check the quality.

### Active Site Prediction

The α-glucosidase protein active sites were studied by means of the “MOE-Site Finder” Module, which computes the possible recognition sites from the 3D atomic coordinates of the protein. The Site Finder module is considered a geometric method, as energy models were not utilized. Rather, the relative positions and accessibility of the protein atoms were targeted followed by a rough classification of chemical types. Once these regions were calculated, dummy atoms were assigned to these sites and later used to make the molecular docking calculation for specified sites.

### Ligand Preparation

The compounds included in our study were all collected from reported literature (12, 23). All these compounds were generated by using the Chembio-Office 2010–2012 and then all these compounds were saved in mol file for the purpose to open these files in MOE and were energy minimized *via* MOE using default parameters.

### Protein Preparation

The modeled structure of the target protein was 3D protonated and then energy minimization was performed by using the MOE software with default parameters.

### Molecular Docking

Molecular docking was performed *via* MOE-dock with most of the default tools with the aim to find the binding interaction of the ligand with the target protein. Ligand was docked into the target site of predicted homology model of the α-glucosidase by mean of MOE-Dock module (v.2011.10), for each ligand 10 conformations were generated. The top-ranked conformation of each ligand was used for detailed study of binding mode.

## Results

### Calculation of Physiochemical Properties

For calculating the physiochemical properties, we used the Expasy-ProtParam server tool. The protein has 584 numbers of amino acids, and its estimated molecular weight was 68,183.3 Da and Theoretical pI: 5.53. The extinction coefficient (EC) of the predicted model was 148,990 at 280 nm, which indicated that at a specific wavelength, how much light was absorbed, EC is in units of m cm^−1^, measured in water at 280 nm. The instability index (II) was computed to be 30.07, which showed the protein was stable. The II more than 40 rated protein as not stable. The hydrophilic character was calculated by the grand average of the hydropathy index value of −0.676, and the aliphatic index was calculated to be 64.28.

### Target-Template Alignment

**Figure 1:**
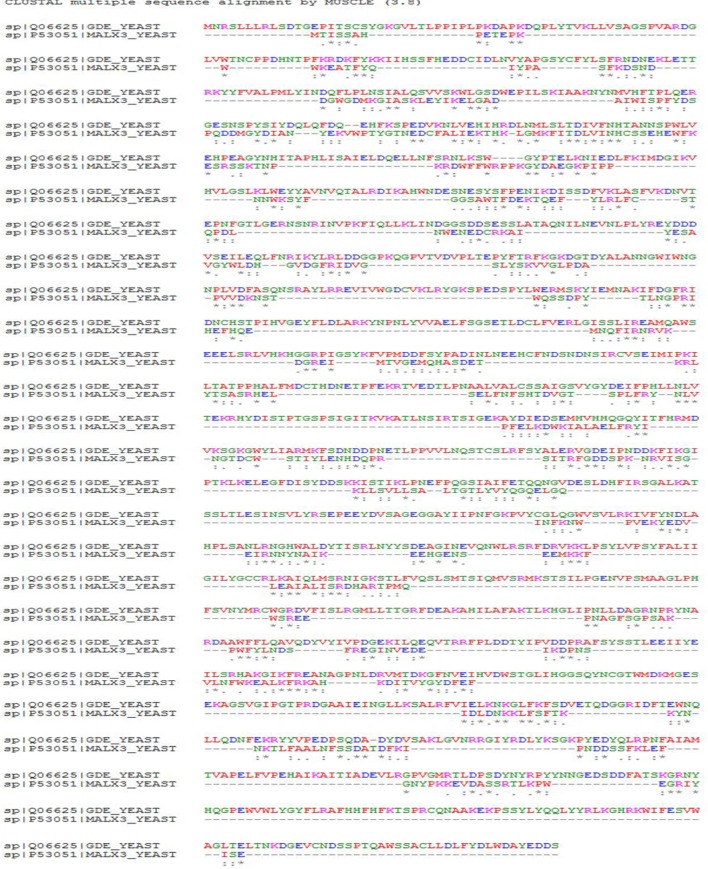
**Target-template alignment by multiple sequence alignment server**.

The MUSCLE server was used for the determination of the alignment of the query sequence to the template. The alignment was made in order for the development of a resulting model. The configuration is presented in Figure 1. In the figure, (*) symbol represents the single entire conserved residues, (-) symbol represents the deleted regions, and (:) and (:) symbols represent the conservation of strong and weak groups correspondingly. Finally, from the evaluation of the results of target-template alignment, it was found that there is sequence homology between α-glucosidase and crystal structure of Isomaltase from *S. cerevisiae* 3A47–A.

### Homology Modeling

For 3D structure modeling, MOE software (MOE 2010–2011) was employed, while used relative protein structure modeling in the following way.

(1) MOE docked the initial partial geometry of target sequence using template structure and used to preserved residue identity.
(2) The residues without specific backbone coordinates were characterized *via* a specific logic insertions and deletions treatment (30).
(3) First, a random order was used for loops modeling. A file of probable candidates were scrutinized using contact energy function based on Boltzmann weighted averaging (31, 32).

**Figure 2:**
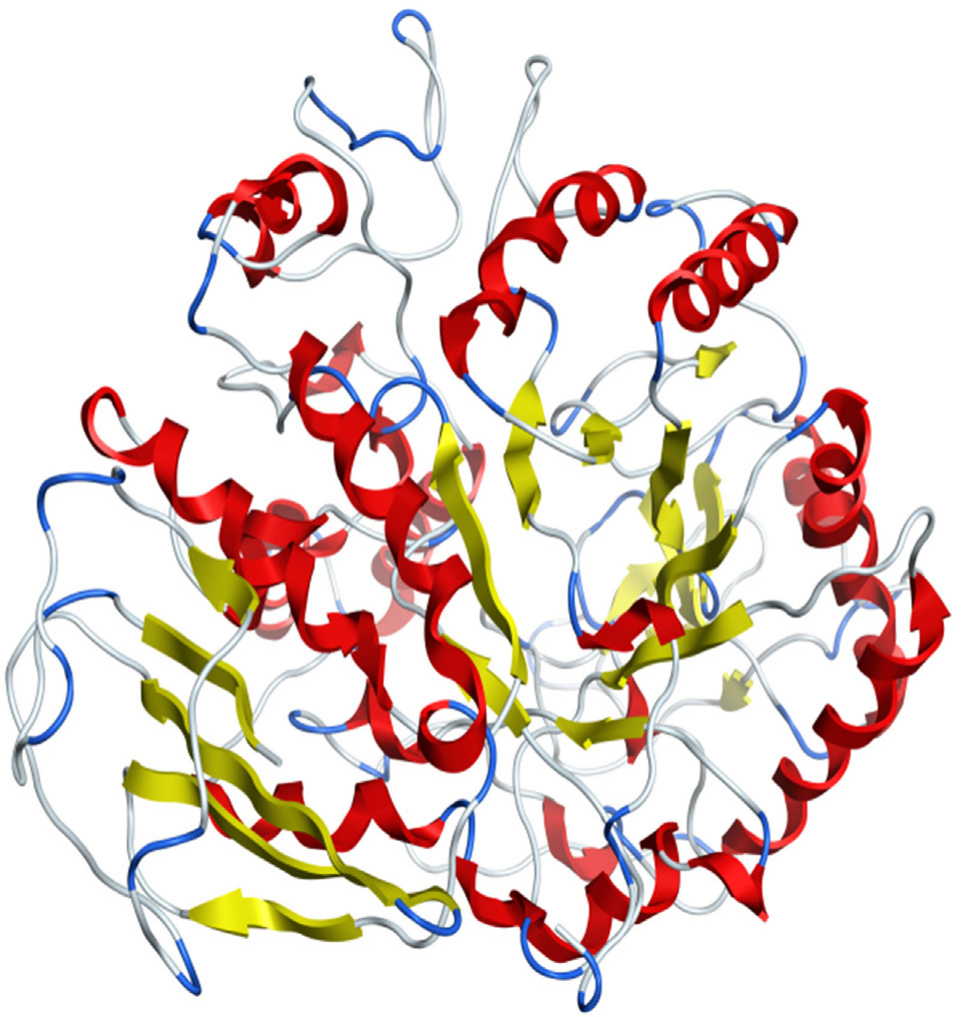
**3D tertiary structure of target**.

**Figure 3:**
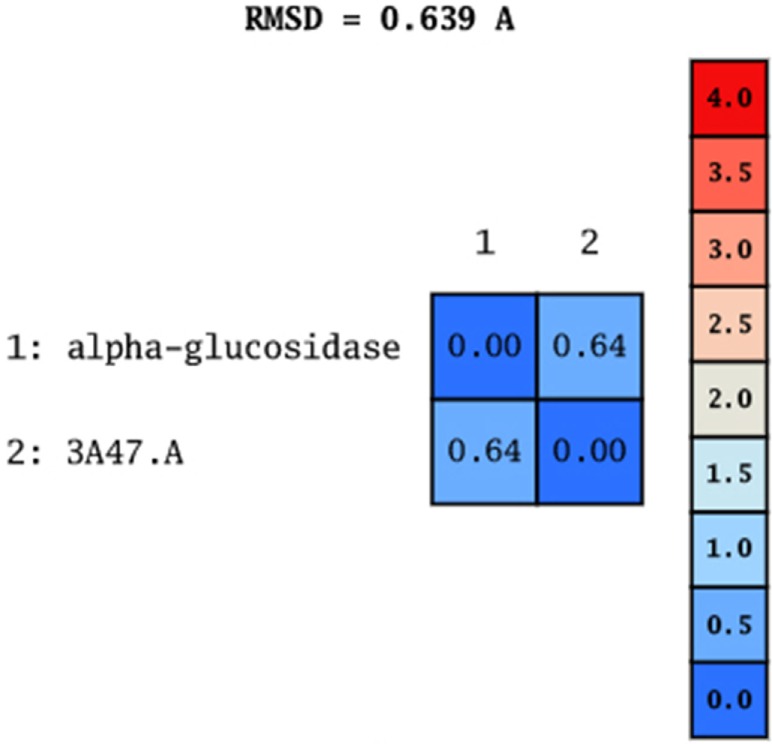
**Root mean square deviation of target and template**.

**Figure 4:**
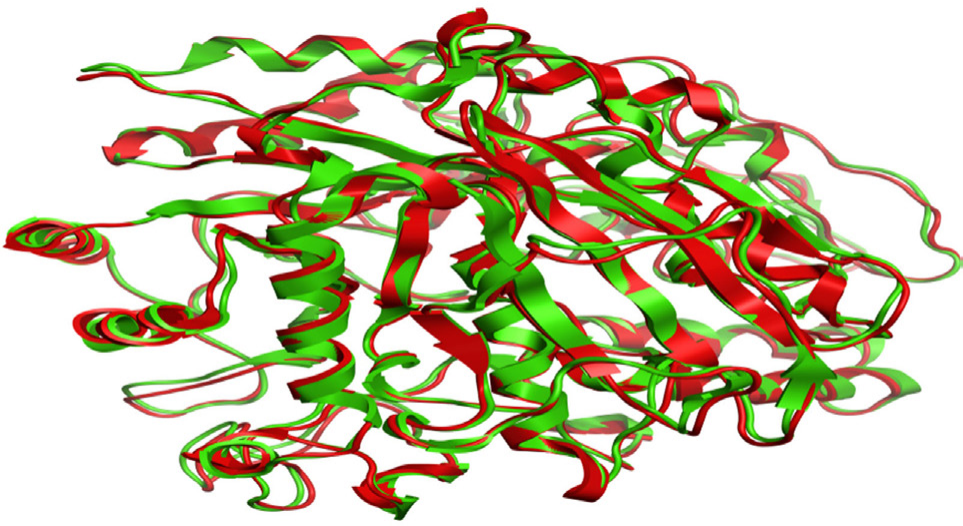
**Superposition of target (red) and template (green)**.

Model was developed while using various tools such as Model Scoring to GB/VI test (33). Force fields to Amber99 used in MOE suggested for protein homology purposes (34). Subsequent to homology modeling, the force field AMBER99 was used to minimize the energy of target structure to 0.05 G and this specifically characterized proteins and nucleic acids. The PDB format was used for resulting structure (Figure 2) having an appropriate identity. The model proposed was superimposed on template structure holding RMSD of 0.639 observing close homology (Figure 3) using the MOE software. The superposition of the target and template are shown in Figure 4.

### Validation of Modeled Structure

**Figure 5:**
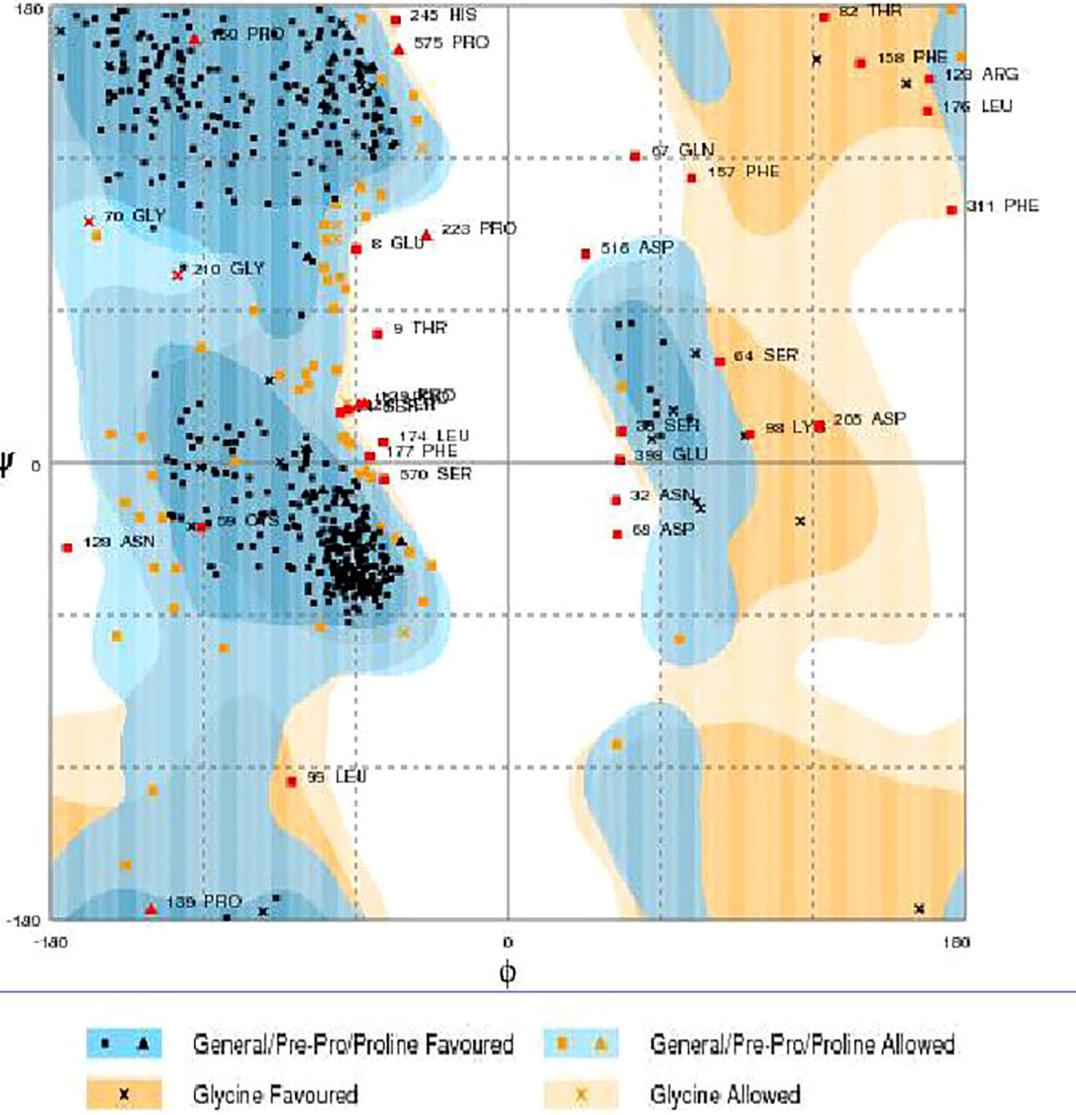
**Ramachandran maps of α-glucosidase produced by RAMPAGE**. Light orange and light blue are allowed regions, while dark orange and dark blue are favored regions.

**Figure 6:**
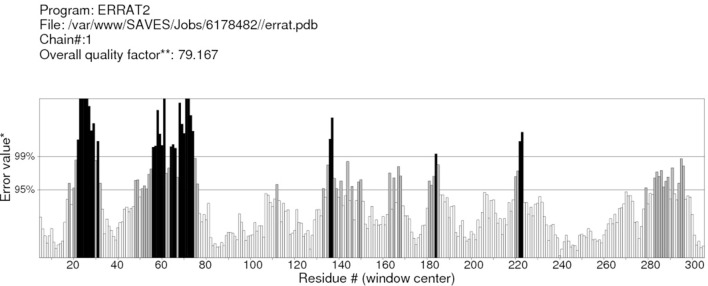
**ERRAT server shows the overall quality of the α-glucosidase**. *On the error axis, two lines are drawn to indicate the confidence with which it is possible to reject regions that exceed that error value. **Expressed as the percentage of the protein for which the calculated error value falls below the 95% rejection limit. Good high resolution structures generally produce values around 95% or higher. For lower resolutions (2.5–3 A), the average overall quality factor is around 91%.

RAMPAGE server explained the stereochemical characters of the 3D structure. Stereochemical evaluation of backbone Phi and Psi dihedral angles (35) is shown in Figure 5. Besides, Ramachandran plot analysis showed that 83.8% components were in the favored region, 10.3% residue in the permissible region, while 5.8% residues in the outlier regions of Ramachandran plot, which indicated that the proposed model is reliable for further studies. The ERRAT server explained the statistics of non-bonded interaction between different atoms, and a score of 50 was generally suitable and for the 3D structure evaluation, the predicted model had quality score 79.167, which indicated that the model was reliable and stable, as shown in Figure 6.

### Active Site Residues

**Figure 7:**
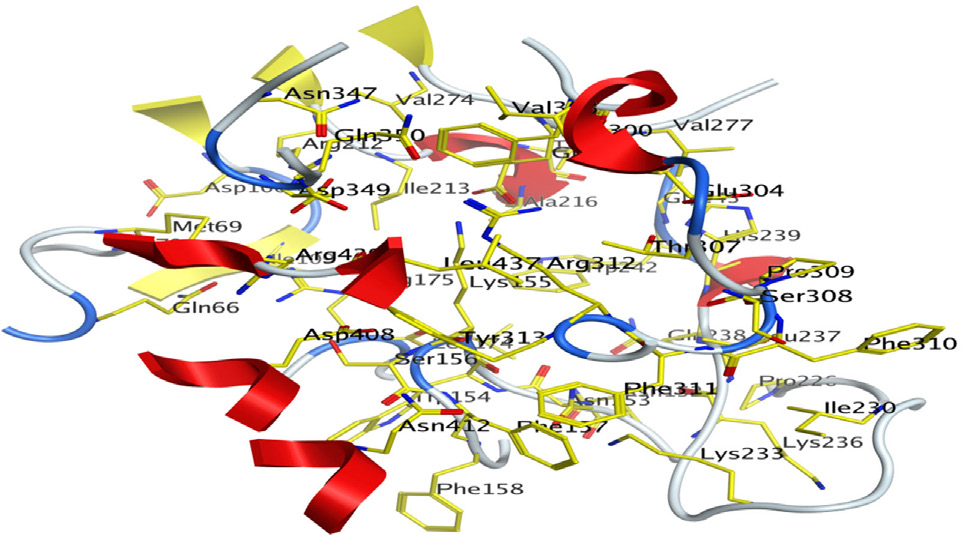
**Predicted binding pocket of α-glucosidase by mean of Molecular Operating Environment Site Finder**.

The Site-Finder Module was utilized for prediction of the ligand-binding site in the predicted model of α-glucosidase showed that GLN66, MET69, ASP106, ILE109, ASN152, ASN153, TRP154, LYS155, SER156, PHE157, PHE158, LEU174, ARG175, ARG212, ILE213, THR215, ALA216, PRO226, ILE230, LYS233, LYS236, LEU237, GLN238, HIS239, TRP242, VAL274, GLU276, VAL277, PHE300, VAL303, GLU304, THR307, SER308, PRO309, PHE310, PHE311, ARG312, TYR313, ASN347, ASP349, GLN350, ASP408, ASN412, LEU437, and ARG439 were found in the binding site of the predicted model of the α-glucosidase, as shown in Figure 7.

### Binding Interaction of Ligands with Target Receptor

In our research studies, we used the 37 known alkaloid inhibitor of α-glucosidase from the previous literature. These known alkaloid inhibitors were tested and proved experimentally from the previous literature (12, 23). In the present research work, we found out the binding interaction and docking scores of these selected compounds with the target protein by molecular docking, as shown in Table 1. These results might be beneficial in the drug designing of the novel and potent inhibitors of the glucosidase.

## Discussion

Compound **17** and compound **24** showed the best docking score i.e., −15.1310 and −14.9192, respectively, among the **37** different alkaloids, and these compounds have good inhibition and docking score nearly similar relative to the reference (standard) ligands, miglitol (−15.4423) and acarbose (−14.7983). These two compounds are most active and showed good interaction with the target protein.

**Figure 8:**
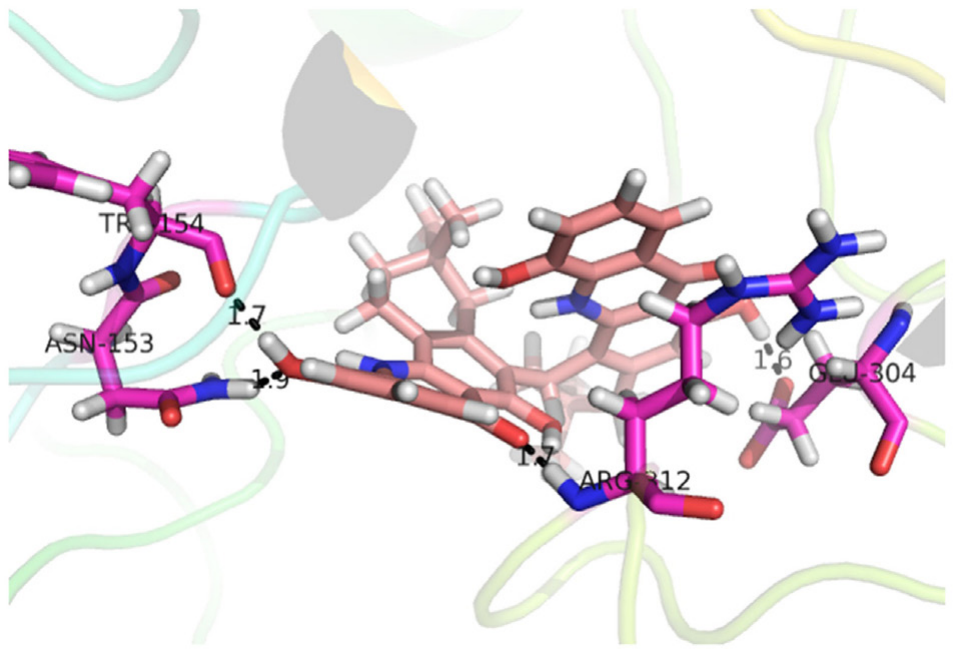
**3D interaction image of most active ligand 17**.

**Figure 9:**
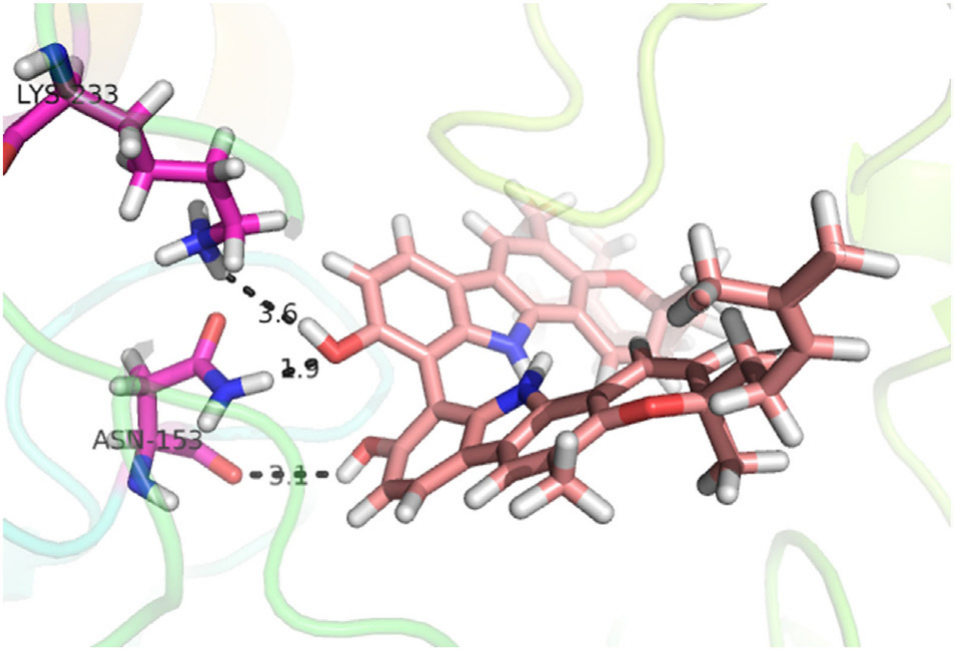
**3D-binding mode of compound 24 with target**.

Compound **17**, the most active ligand, formed three hydrogen bonds, one hydrophobic interaction, and one arene cation interaction with the Asn153, Arg312, Glu304, Trp154, and Lys155 active amino acid residues, respectively. Lys155 formed an arene cation interaction with the benzene ring of the phenol moiety of the ligand. Glu304 was observed making a hydrogen bond with the hydrogen atom of the OH group of another phenol group of the same ligand. Arg312 and Asn153 formed hydrogen bonds with the oxygen atom double bonded with the piperidine moiety of the ligand and oxygen atom of −OH moiety of the phenol of dihydroxy-3,3-dimethyl-1,2,3,4-tetrahydrobenzo[c]acridin-7(12H)-one, respectively, of the same inhibitor. Trp154 showed a hydrophobic interaction with the H atom of the −OH group of the dihydroxy-3,3-dimethyl-1,2,3,4-tetrahydrobenzo[c]acridin-7(12H)-one moiety. The 3D interaction of the ligand with receptor was shown in Figure 8. Similarly, these results were in absoulte agreement with the *in vitro* α-glucisade inhibitory activity already reported. Moreover, it also caused marked free radical scavenging effect (20). Compound **24** is the second most active ligand and interacted with the Asn153 and Lys233 and was observed making three hydrogen bonds. Asn153 formed H-bonds with the hydrogen atom of OH group and with the oxygen atom of another −OH group of the same ligand. Lys233 was observed making an H-bond (hydrogen bond) with the −O atom (oxygen atom) of the hydroxyl moiety of the inhibitor, as shown in Figure 9. The docking results are consistant with the *in vitro* α-glucisade inhibitory activity already reported, agumented by its comprehensive antioxidant effects (23).

**Figure 10:**
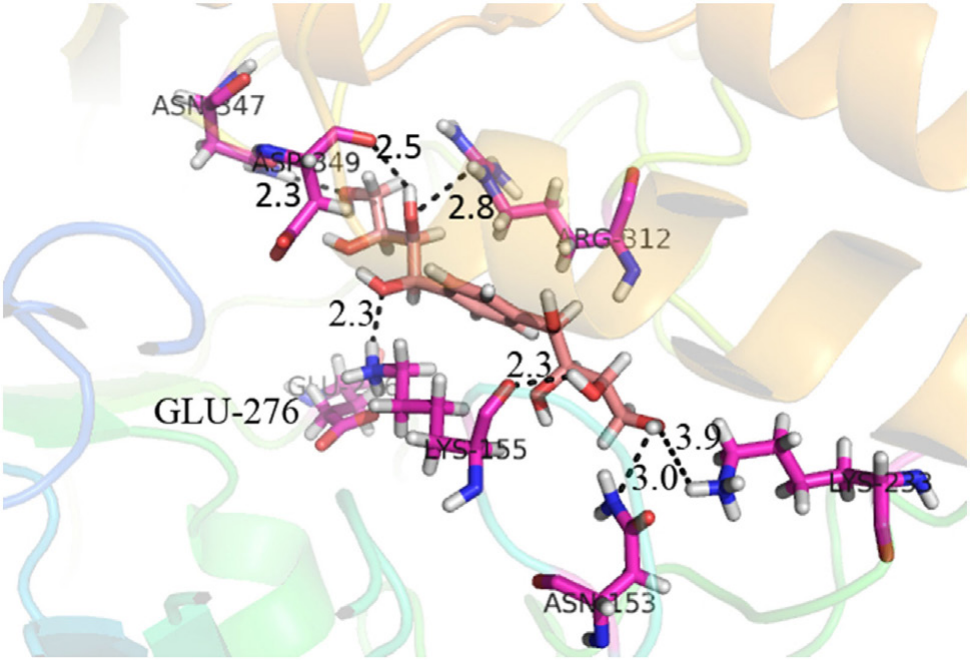
**3D interaction of compound 37**.

Compound **37** has moderate good docking score −10.4884 and have the best interaction with active residues. It formed five hydrogen bonds with the Asn153, Lys155, Lys233, Glu276, and Asn347, a hydrophobic interaction with the Asp349 and arene cation interaction with the Arg312. Arg312 made an arene cation interaction with the phenylene moiety of the ligand. Lys155 and Glu276 formed hydrogen bonds with the oxygen atom and hydrogen atom of the butane-1-ol. Asn153, Asn347, and Lys233 formed hydrogen bond interactions with the oxygen atom of the hydroxyl group (−OH) of the butane-4-ol. Asp349 formed ahydrophobic interaction with the hydrogen atom of the −OH group of the butane-2-ol moiety of 1,1′-(1,4-phenylene)-bis(butane-1,2,3,4-tetraol) shown in Figure 10.

**Figure 11:**
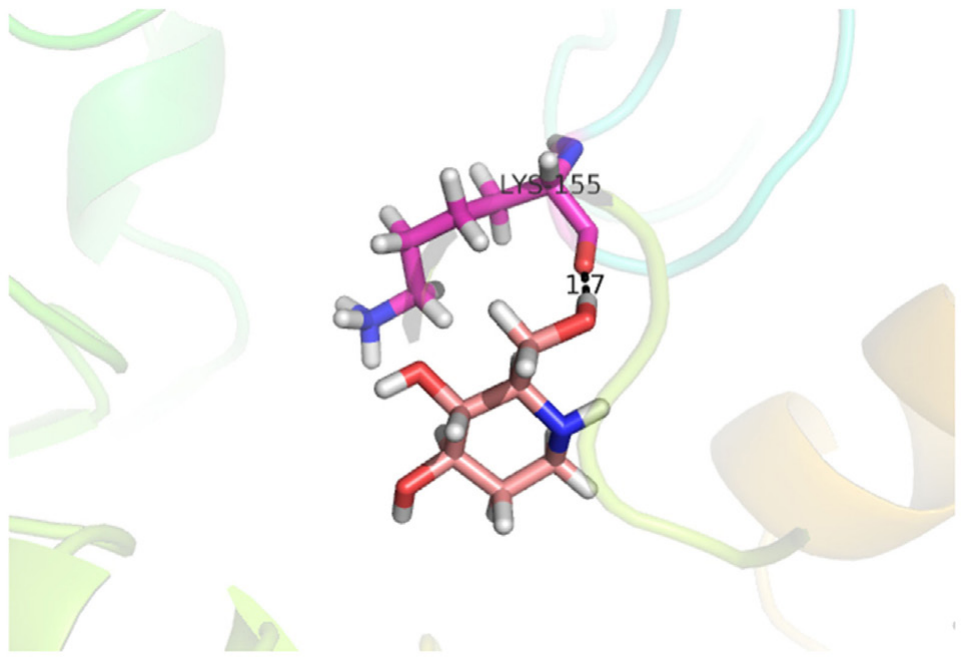
**Docked conformation of compound 33 with receptor**.

**Figure 12:**
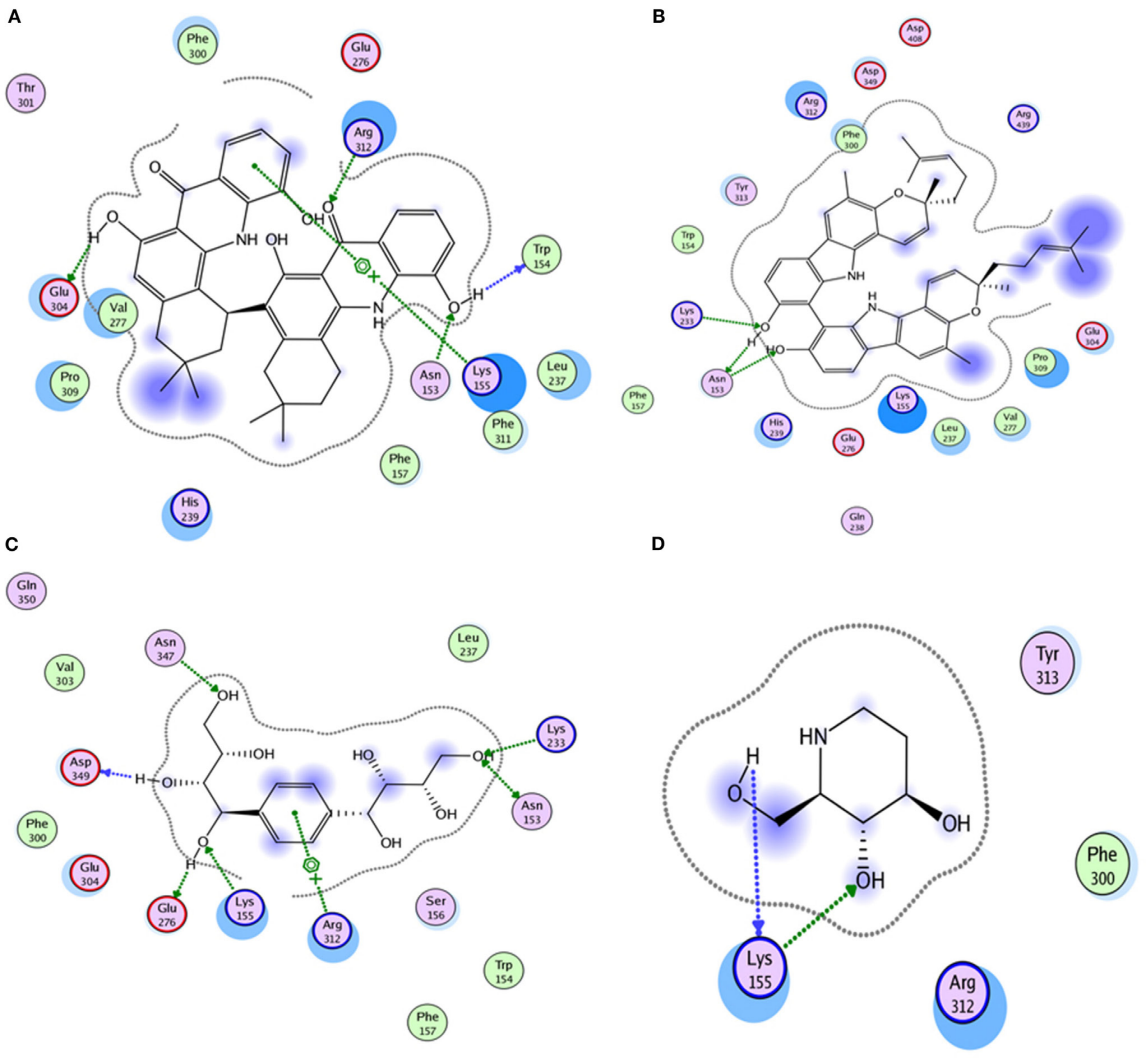
**The figure shows the 2D images of the docked conformations of the ligands with the active residues (A) 2D image of compound 17, (B) 2D image of compound 24, (C) 2D image of compound 37, and (D) 2D image of compound 33**.

Compound **33** had low docking score −5.8070 and showed a low number of interaction with the active residues, i.e., formed one hydrogen bond and one hydrophobic interaction with the Lys155, as given in Figure 11. Lys155 made a hydrogen bond with the oxygen atom of OH group, and Lys155 interacted with the hydrogen atom of another −OH moiety of the (2R,3R,4R)-2-(hydroxymethyl)piperidine-3,4-diol. Figure 12 showed the two-dimensional interaction images of the compounds with the target protein.

## Conclusion

The molecular docking was made to recognize the binding interactions of these reported compounds with the receptor. We examined the interaction of inhibitors to that of our target receptor. The molecular docking study indicates good docking score and binding mode and thus showed therapeutic potential of these compounds on α-glucosidase inhibition. Compound **17** (oriciacridone F) and **24** (O-methylmahanine) demonstrated marked interaction with active site residues, which are also correlated with the reported IC_50_ values and, thus, might be the best candidates for the discovery of novel α-glucosidase inhibitors, after *in vivo* efficacy, safety, and clinical studies.

Moreover, the rest of alkaloids with good receptor interaction could be lead compounds and therefore need further studies in terms of synthesis, structural relationship activity followed by testing in various *in vitro* and *in vivo* testing.

## Author Contributions

MZ, AK, and ML carried out the *in silico* studies and initial draft of the manuscript. AR assisted in molecular docking of test compounds. HK supervised the entire study and finalized the draft.

## Conflict of Interest Statement

The authors declare that the research was conducted in the absence of any commercial or financial relationships that could be construed as a potential conflict of interest.

## Footnotes

^1^http://www.bioplek.org/animaties/enzymen/maltase2.html.

^2^http://www.uniprot.org/.

^3^http://www.ebi.ac.uk/Tools/msa/muscle/.
